# Phyto-nano-hybrids of Ag-CuO particles for antibacterial activity against drug-resistant pathogens

**DOI:** 10.1186/s43141-020-00068-0

**Published:** 2020-09-21

**Authors:** Syed Baker, Perianova Olga, Rukosueva Tatiana, Potkina Nadezhda, Garkusha Tatyana, Rukovets Tatyana, Elena Saveleva, Khokhlova Olga, Gudkova Elizaveta, Gildeeva Karina, Udegova Ekaterina, Sergeenako Anastasia, Putintseva Margarita

**Affiliations:** 1 Department of Microbiology, Prof. V.F. Voino-Yasenetsky Krasnoyarsk State Medical University, Partizana-Zheleznyaka Street, 1, Krasnoyarsk, Siberia Russian Federation 660022; 2 Krasnoyarsk State Territorial Bureau of Pathology, Partizana Zheleznyaka str. 3 “D”, Krasnoyarsk City, Russian Federation 660022; 3 Department of Biochemistry, Prof. V.F. Voino-Yasenetsky Krasnoyarsk State Medical University, ul Partizana Zheleznyaka, 1, Krasnoyarsk, Russian Federation 660021; 4 Department of Pharmaceutical technology and Pharmacognos, Prof. V.F. Voino-Yasenetsky Krasnoyarsk State Medical University, Partizana-Zheleznyaka street, 1, Krasnoyarsk, Siberia Russian Federation 660022

**Keywords:** Phytogenic, Nano-hybrid, Silver nanoparticles, Copper oxide nanoparticles, Multi-drug resistance, Antibacterial properties

## Abstract

**Background:**

The present study reports the antibacterial potential of phyto-nano-hybrid particles Ag-CuO (silver-copper oxide) against drug-resistant pathogens isolated from a Russian hospital in Krasnoyarsk, Siberia. The synthesis of nano-hybrid was achieved by phytogenic source by using leaves of *Murraya koenigii*. The nano-hybrid particles were well characterized using hyphenated techniques and results of the antibacterial assay was tabulated.

**Results:**

The UV-visible spectra displayed absorption at 420 nm with the shoulder peak at 355 nm indicating the hybridization. The FTIR analysis revealed the presence of phenol, amine, methyl, carbohydrate and aromatic as major functional groups. The XRD analysis revealed the presence of Bragg’s intensities at 2 theta angle depicting the crystalline nature of Ag-CuO nano-hybrid. The TEM analysis displayed the polydispered properties of Ag-CuO nano-hybrid with the size in the range of 60–80 nm exhibiting different shapes ranging from spherical, rod and oval. The antibacterial activity of Ag-CuO nano-hybrid was tested against multidrug-resistant pathogens that resulted in highest activity against *P*. *aeruginosa* strain with an inhibition zone of 14 mm in diameter. The MIC concentrations ranged from 0.3125 to 2.5 μg/ml and broth dilution assay displayed dose-dependent properties of Ag-CuO nano-hybrid particles.

**Conclusion:**

The obtained results are interesting to report the preliminary insight to develop biocompatible hybrid particles to combat drug-resistant pathogens. The developed nano-hybrid particles displayed activity against all the test pathogens investigated against both Gram-positive and Gram-negative bacteria. Thus, the study forms preliminary investigation to report nano-hybrid particles as broad spectrum antibacterial agents.

## Background

The implementation of nanoscience has led to considerable advancements in the development of novel structures for specific activities [1]. In recent years, construction of hybrid structures is of great interest since they efficiently perform desired activity [2]. The process of hybridization can be of different types based on the role and type of participating materials [3]. The utilization of evolved nano-hybrid complex can substantially improve the existing system or application. Metallic nanoparticles have proven greater efficiency to act as potent antibacterial agents in the recent years [4]. The efficacy of metallic nanoparticles to inhibit or suppress the growth of pathogenic bacteria can be attributed to their unique physicochemical properties [5, 6]. In the current scenario, there is an urgent need to develop novel or alternative antibacterial agents to combat drug resistance which has posed deleterious effects to increase mortality and morbidity rates [7]. According to the World Health Organization (WHO), antimicrobial infections will be magnified by 2050 and is expected to reach 10 million deaths due to antimicrobial resistance [8]. The situation is deteriorating with time and paucity of effective antibiotics is creating an alarming situation. There have been considerable efforts to design novel molecules to combat drug resistance. One among such candidates includes synthetic bimetallic nanoparticles bearing antibacterial activity [9]. In comparison to the monometallic nanoparticles, bimetallic nanoparticles exhibit distinct characteristics which have created appealing trends among the scientific researcher to design bimetallic hybrid nanostructures [10]. The bimetallic nanoparticles differ physically and chemically such as surface area, optical characteristics, magnetic properties and functional bioavailability with respect to their counterparts [11]. These properties are more predominant in comparison with individual nanostructures. Based on these key fundamental facts, hybrid nanostructures are widely in practice such as diagnostic kits, bio-functionalization, electronic devices, high-storage capacity and bioanalytics [12]. Nanostructures like silver-copper oxide hybrid have significant properties due to their radical character and are capable of producing hydrogen peroxide which in turn interfere the metabolic process of the bacterium [13]. As monometallic nanoparticles, copper oxide nanoparticles are thermodynamically more stable and usage of copper can be traced from history which was used in curing wounds and in drinking water treatments [14]. In recent years, copper is part of healthcare systems in sanitizing, developing gas sensors, semi-conductors, solar cells, biosensors, paint industries and antimicrobial agent [15]. Copper is reported to have different actions on targeted pathogens and also mediate the contact killing. Similarly, application of silver for such purposes is documented in traditional records as well [16]. Among the metallic nanoparticles, silver nanoparticles are considered to be one of the most populated subjects of investigation. Lately, there are also reports of pathogenic bacteria developing resistant to silver component as the drug-resistant pathogens engineer their metabolic process to mask the effect of silver [17]. Synthesizing the hybrid nanostructure from biogenic sources to overcome the limitation of conventional routes has gained substantial attention in recent years [18]. The biological molecules such as polysaccharides, proteins, vitamins and phyto-components are employed as reducing agent to synthesize desired nanostructures [19, 20]. These biological compounds act as capping agents which are reported to enhance the desired activities [21]. One such noteworthy application of hybrid nanostructures can be cited as excellent antibacterial agent. In the present investigation, the nano-hybrid was synthesized using *Murraya koenigii* as phytogenic source and used for antibacterial activity against drug-resistant pathogens. These pathogens are clinically isolated from the patients suffering from myriad infection and reported to cause secondary infections.

## Methods

### Preparation of plant extract

The leaves of *Murraya koenigii* were freshly collected from the abundant growing area of Mysore region, Karnataka, southern part of India. The plant was collected and identified at Herbal Drug Technological laboratory, University of Mysore. The identification was carried out under the guidance of Prof. Satish. The morphological characteristics were matched with the culture collection centre which is available publicly. Currently, the accession number is not available owing to the renewal of university norms. After the identification process, the leaves were subjected to sun drying for 1 week to remove aqueous components from the healthy leaves as the presence of water content can result in favouring microbial contamination. The leave samples were weighed, 20 g of healthy leaves of *Murraya koenigii* were boiled in 200 ml of sterile distilled water for 60 min. The aqueous extract obtained was filtered aqueously and used for the synthesis of nanoparticles as per the previous report [21].

### Synthesis of nano-hybrid particles

Synthesis of nano-hybrid was carried out by preparing the mixture of 1 mM silver nitrate solution with 10 mM copper sulphate solution in 1:1 ratio. The mixture was treated with aqueous plant extract and was stirred on heating mantle at temperature 90 °C on water bath for 2 h. The pH of the reaction mixture was increased with the addition of 1 mM NaOH solution until the reaction mixture changes. The mixture was then centrifuge at 18,000 rpm for 30 min and washed with sterile distilled water [21]. The pellet was subjected to characterization using analytical tools.

### Characterization of nano-hybrid particles

The synthesized nano-hybrid particles were characterized using UV-visible spectroscopy and maximum absorbance was recorded. The sample was subjected to vacuum pressure to remove the water content. The dried sample was layered onto the grid and morphological characteristic of nano-hybrid particles were studied with TEM model Zeiss Libra 120 PLUS. The instrument was equipped with TRS camera at Dual speed 220-V 50–60-Hz operating with Program—Olympus iTEM. The nano-hybrid particles were recorded and measured to obtain average size. The possible role of phyto-components to mediate the synthesis of nano-hybrid particles was studied using Fourier transform infrared spectroscopy (FTIR) analysis recorded on Shimadzu IRAffinity-1 spectrometer. The crystalline nature was determined using X-ray diffraction (XRD) analysis out according to the protocol described by Baker et al. [6], wherein the dried sample was coated on the XRD grid and spectra were recorded with Rigaku Miniflex operating at 30-kV voltage. The spectral scan was performed between 0 and 80° at 2 theta angle.

### Antibacterial activity of nano-hybrid particles

The antibacterial activity was initially determined via well diffusion assay against the test pathogens (*A*. *baumannii* strain 211, *A*. *baumannii* strain 210, *P*. *aeruginosa* strain 40, *P*. *aeruginosa* strain 215, *K*. *pneumoniae* strain 104, Methicillin-resistant *Staphylococcus aureus*, *E*. *coli* strain 55). The selected bacterial pathogens were procured from the Department of Microbiology culture collection centre of Prof. V.F. Voino-Yasenetsky Krasnoyarsk State Medical University, Krasnoyarsk. These pathogens were isolated from the clinical samples and characterized to assign different accession codes as per the collection centre manual. The standard inoculum was prepared with McFarland dilution methods in accordance with Clinical and Laboratory Standards Institute (CLSI) guidelines [15]. The antibacterial properties were determined using well diffusion assay with Mueller Hinton agar plates which were swabbed with inoculum, agar wells were punched and nano-hybrid particles (5 mg/ml concentration) were added to the well during which the diffusion of the nano-hybrid particles take place and incubated at 37 °C overnight. The activity was measured as a zone of inhibition across the well using Hi-media standard measuring scale.

### Antibacterial activity using broth dilution assay

The activity was also evaluated using broth dilution assay with increasing concentration of nano-hybrid particles from 0 to 100 μg/ml added into test tube containing 10 ml nutrient broth seeded with 10^6^ colony forming units. The growth pattern was recorded after the incubation period using UV-visible spectrophotometer at 600 nm [15].

### Antibacterial activity using minimal inhibitory concentration assay

The minimum inhibitory concentration was carried out as per our previous report. In brief, the minimal inhibitory concentration (MIC) plates were prepared by adding 100 μL of test nano-hybrid particles followed by the addition of 50 μL nutrient broth onto the first well which was serially diluted using multi-channel pipette. As a growth indicator, 10 μL resazurin was added to each well. The final volume of the broth was maintained by adding 30 μL isosensitized broth to each well. Later, 10 μL of test bacterial suspension was added to each well. The plates were incubated at static condition at 37 °C for 18 to 24 h. In order to determine the lowest concentration of nanoparticles to inhibit the growth of test pathogens, minimal inhibitory concentration was carried out using resazurin as growth indicator dye [21]. Resazurin acts as an oxidation-reduction indicator with blue colour in nature and becomes pink upon reduction by oxidoreductase enzyme. This property was used for visual observation to provide the growth indication.

## Results

**Fig. 1:**
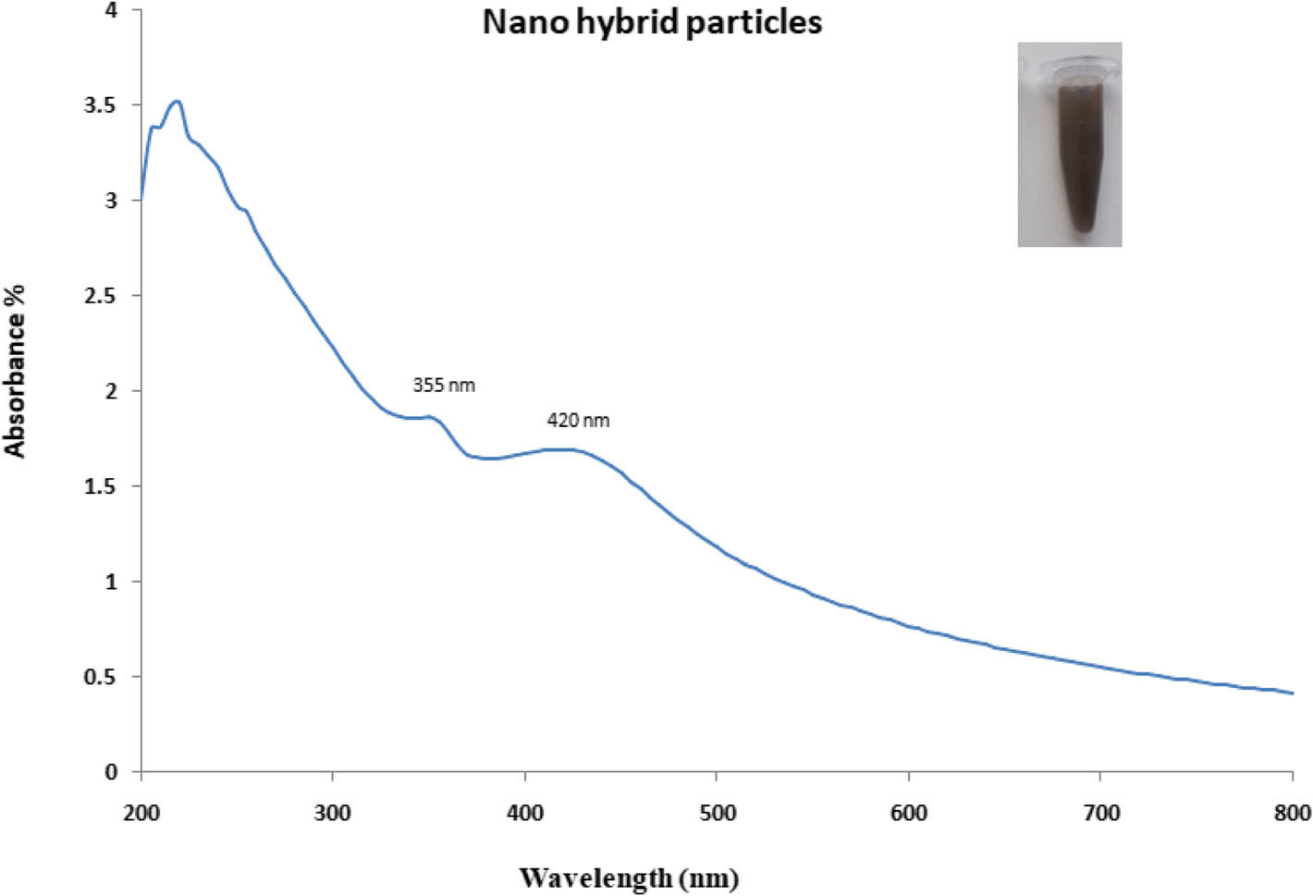
UV spectrum of nano hybrid particles (Ag-CuO)

In the present study, nano-hybrid particles were synthesized and evaluated for antibacterial properties against the clinical isolates which displayed resistance to most of the standard antibiotics (Table 1). The synthesis of nano-hybrid particles was initially confirmed with the change in the colour of the reaction mixture (Fig. 1, inset). The nano-hybrid particles were centrifuged and the pellet was further used for investigating the characteristics using analytical tools.

**Table 1:** Antibiotic profile of test pathogens

Strains	CFP	CAZ	FEP	IMP	MEM	TIM	GEN	TOB	AK
**PA-40**	R	S	S	R	R	R	S	S	S
**PA-215**	S	S	S	R	R	R	S	S	S
**A.B-210**	R	R	R	R	R	R	R	S	R
**A.B-211**	R	R	R	S	R	R	R	S	R
**K.P-104**	R	R	S	R	R	R	R	R	R
**E.C-55**	R	R	S	S	R	S	R	R	R

*CFP*, cefoperazone; *CAZ*, ceftazidime; *FEP*, cefepim; *IMP*, imipenem; *MEM*, meropenem; *TIM*, ticarcillin; *GEN*, gentamicin; *TOB*, tobramicin; *AK*, amicacin; *PA*-*40*, *P*. *aeruginosa* 40; *PA*-*215*, *P*. *aeruginosa strain* 215; *A*.*B*-*210*, *A*. *baumannii* strain 210; *A*.*B*-*211*, *A*. *baumannii* strain 211; *K*.*P*-*104*, *K*. *pneumoniae* strain 104; *E*.*C*-*55*, *E*. *coli* strain 55

### Characterization of nano-hybrid particles

**Fig. 2:**
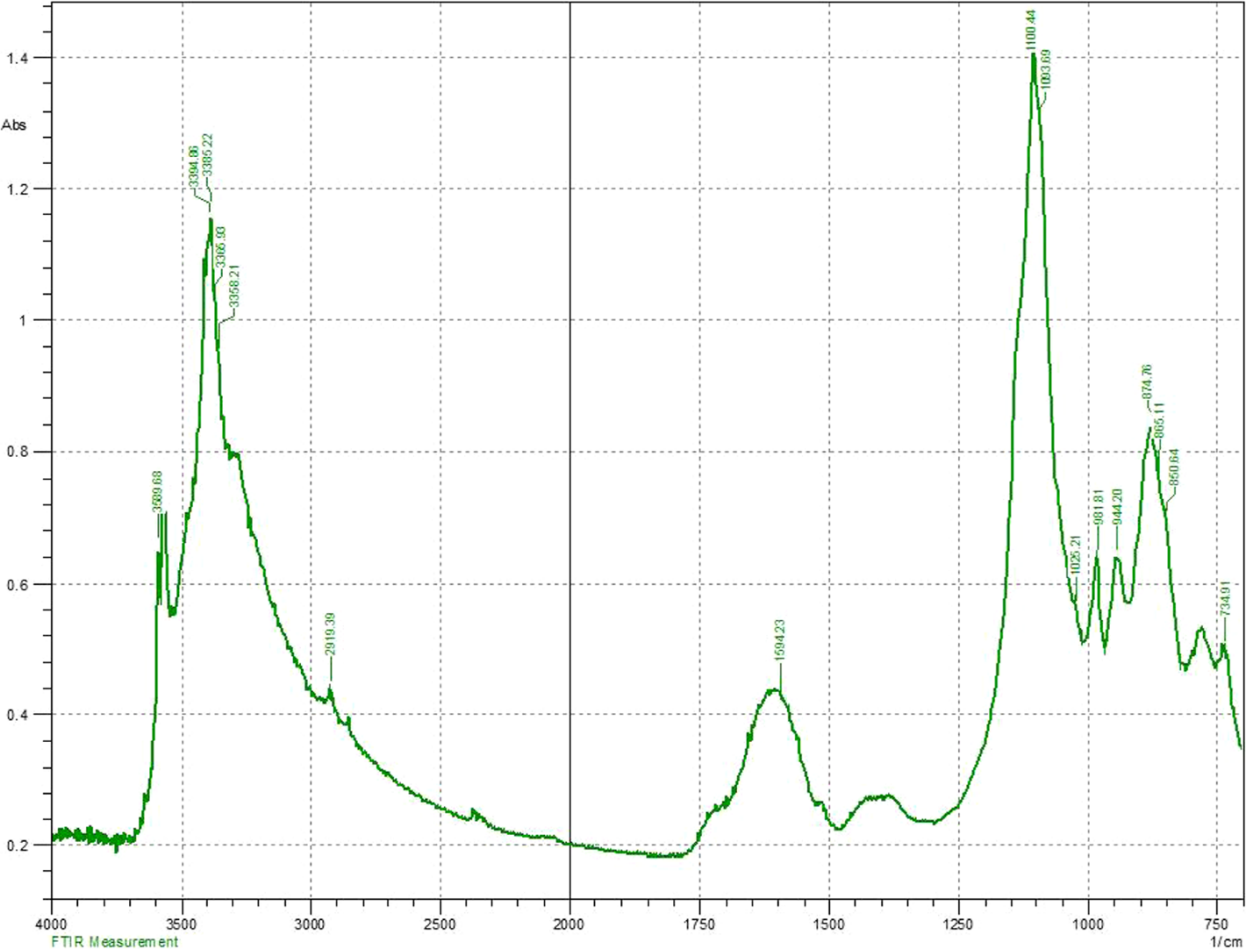
FTIR analysis of nano hybrid particles (Ag-CuO)

**Fig. 3:**
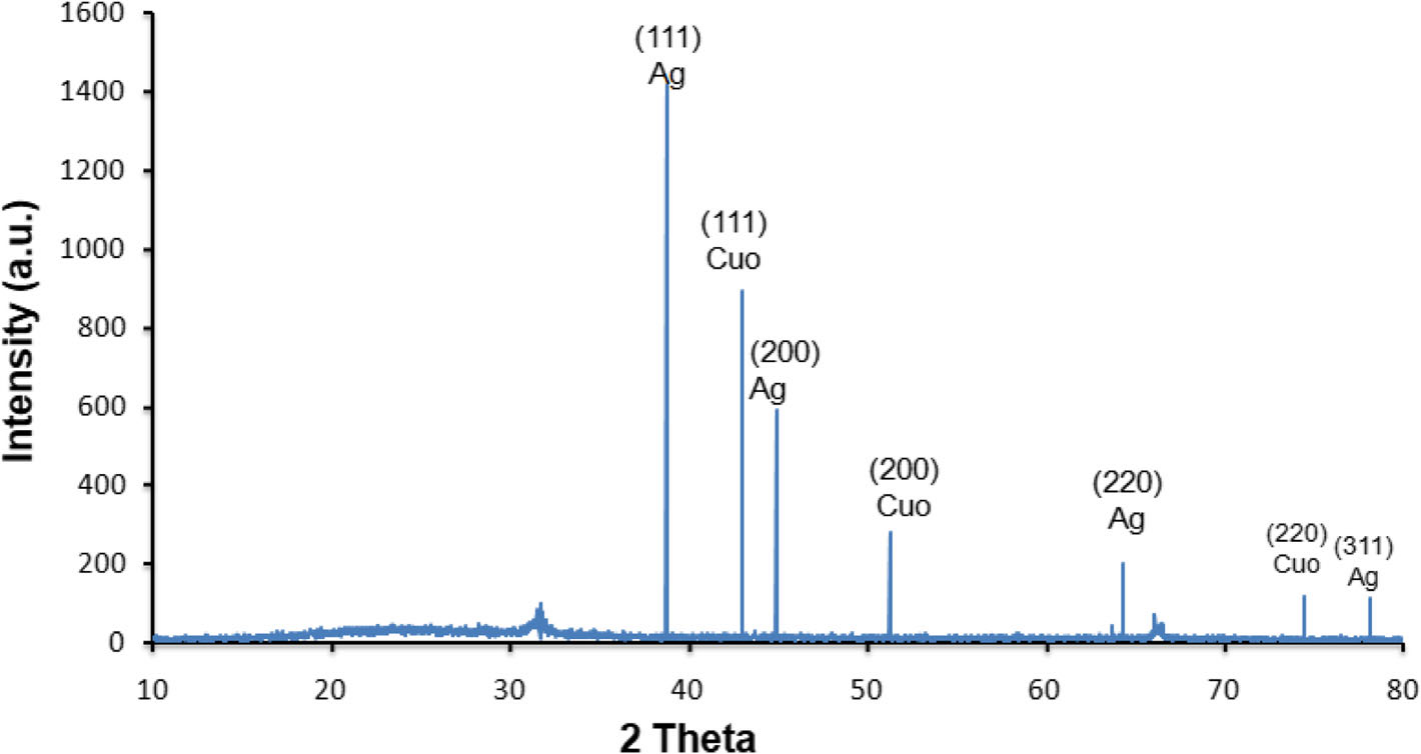
XRD analysis of nano hybrid particles (Ag-CuO)

**Fig. 4:**
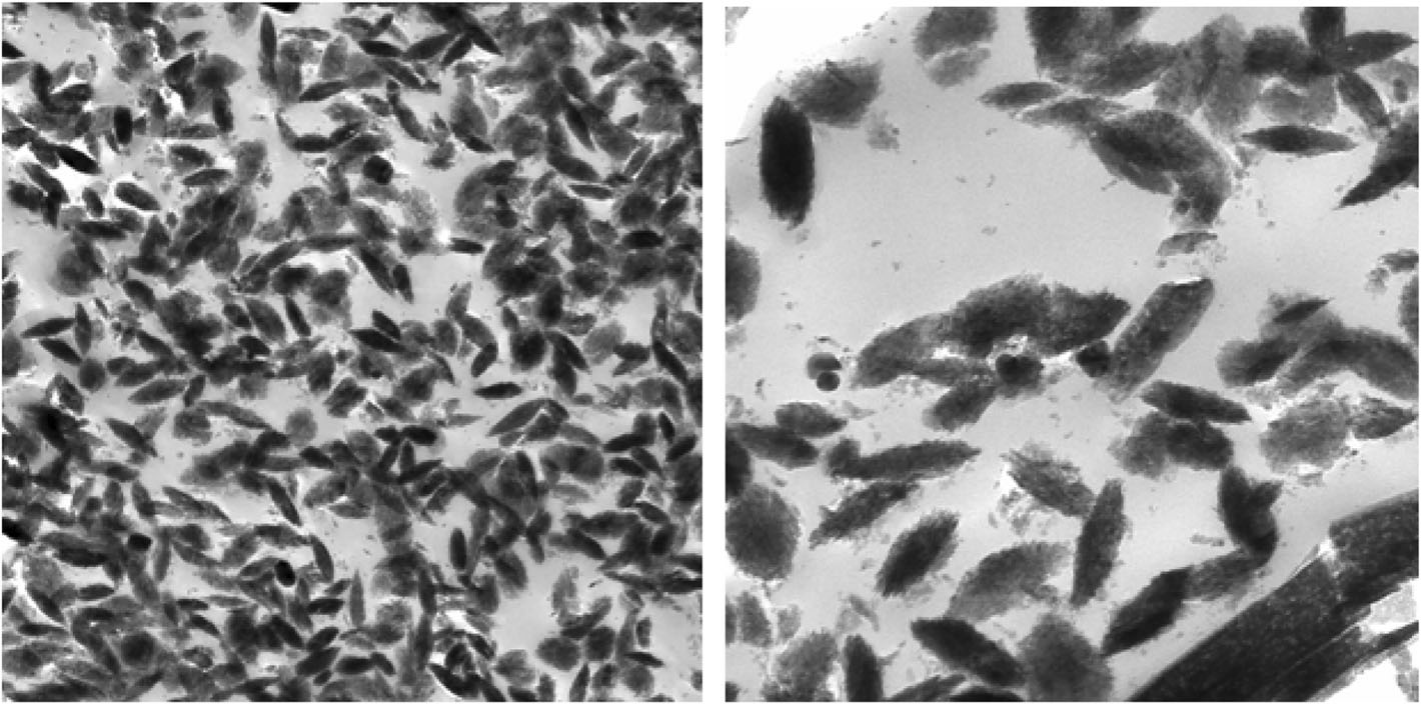
TEM analysis of nano hybrid particles (Ag-CuO)

The synthesis of nano-hybrid nanoparticles was confirmed with characteristic UV absorbance peak at 420 nm along with additional shoulder peak at 355 nm (Fig. 1). The influence of temperature displayed maximum synthesis at elevated temperature 90 °C. The role of pH also favoured the synthesis process with alkaline pH 12 changing the colour of the reaction mixture indicating the synthesis process. The FTIR analysis revealed the presence of phenol, amine, methyl, carbohydrate and aromatic as major functional groups (Fig. 2). The Bragg’s intensities at 2 theta angle confirmed the crystalline nature of the nano-hybrid particles (Fig. 3). The additional peaks might be due to the associated phyto-components. The TEM analysis displayed the majority of nano-hybrid particles to be oval in shape and the average size was calculated by counting nano-hybrid particles which revealed 60 to 80 nm in size (Fig. 4). Few of the nano-hybrid nanoparticles were even bigger than 100 nm which might be due to the hybridization between silver-copper oxide (Ag-CuO) thus forming larger particles.

### Antibacterial activity of nanoparticles

**Fig. 5:**
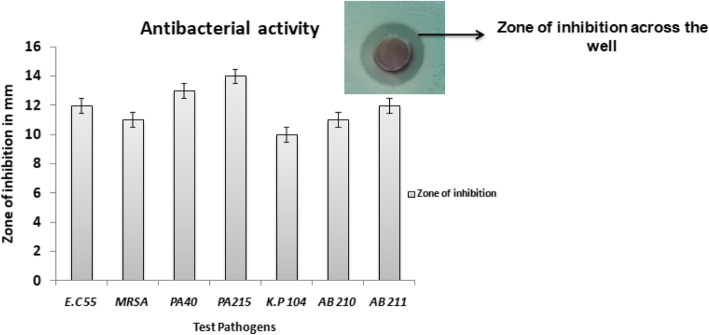
Antibacterial activity of nano hybrid particles (Ag-CuO)

**Fig. 6:**
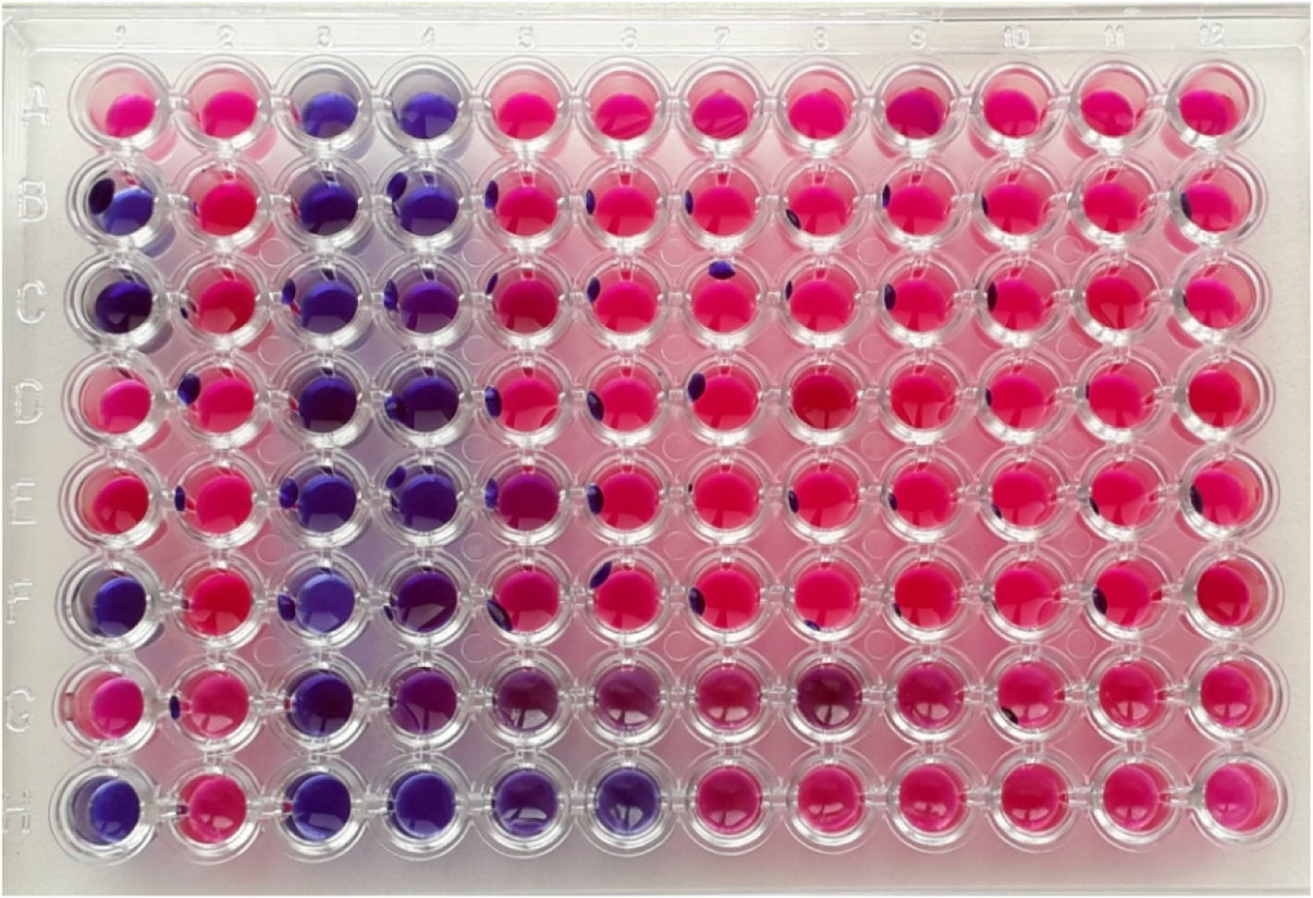
Antibacterial activity using MIC of nano hybrid particles (Ag-CuO)

In the present study, the nano-hybrid particles synthesized from *Murraya koenigii* displayed antibacterial activity against all the test pathogens. The antibacterial activity tested against targeted pathogen viz., *A*. *baumannii* strain 211, *A*. *baumannii* strain 210, *P*. *aeruginosa* strain 40, *P* .*aeruginosa* strain 215, *K*. *pneumoniae* strain 104, Methicillin-resistant *Staphylococcus aureus* and *E*. *coli* strain 55. The activity was observed with well diffusion assay indicating the clear zone of inhibition across the well to suppress the growth of test pathogens (Fig. 5). The activity was further confirmed with broth dilution assay which displayed activity based on the concentration of nanoparticles. As the concentration was increased from 0 to 100 μg/ml, the activity was also found to increase indicating dose-dependent property of the nano-hybrid particles. The activity was also determined with minimal inhibitory concentration to determine the lowest concentration of nanoparticles to suppress the growth of the test pathogens. The MIC concentrations of the test nano-hybrid particles ranged from 0.3125 to 2.5 μg/ml (Fig. 6). The results obtained in the present investigation displayed the role of nano-hybrid particles to act upon the drug-resistant pathogens which belong to the drug-resistant communities.

## Discussion

The synthesis of nano-hybrid particles was carried out using aqueous extract from *Murraya koenigii*. The selection of plant was based on the scientific traditional records on its usage [22]. The *Murraya koenigii* is considered one of the traditional herb-bearing therapeutic index and is being used as species to add aroma in food [23]. It is reported to have various health benefits as per the scientific records [24]. Based on these facts and consideration, *Murraya koenigii* was selected as the subject of investigation. The reduction process was well determined with spectroscopy owing to the excitation of electron on the surface of the nano-hybrid particles to conduct surface plasmon resonance [25]. This can be cited in the previously reported scientific investigations [26]. The physical parameters play very important roles in the rapid and maximum synthesis of nano-hybrid particles [27]. In the present investigation, elevated temperature coupled with alkaline pH influenced the synthesis. The obtained results are in accordance with the previously reported investigations [28]. The role of phyto-components acting as reducing agent to mediate the synthesis has been well demonstrated with sporadic scientific literatures [19]. The study on *Murraya koenigii* has already revealed the presence of alkaloids, flavonoids, steroids and carbohydrates which might be responsible for the synthesis of nanomaterials [24]. In the present study, presence of phenol, amine, methyl, carbohydrates and aromatic as major functional groups centred at vibrational peaks. The use of FTIR analysis is one of the most convenient tools to determine the functional groups associated with the nanoparticles [20]. The obtained results are inconsistent with the previously reported articles [21].

The Bragg’s intensities at 2 theta angle correspond to face centric cube of Ag and CuO nanoparticles. The obtained results are in accordance with the earlier scientific study [29, 30] The morphological characteristics was determined using TEM analysis which resulted in polydispersity of nano-hybrid particles which was similar to most of the earlier reports of plant-mediated synthesis and coincide with the earlier reports of bimetallic synthesis of silver-copper nanoparticles [31]. The antibacterial potential of nano-hybrid particles displayed significant activity against all the test pathogens. There are earlier reports on solvent extracts of *Murraya koenigii* displaying antibacterial activity which are well documented [32]. It will be interesting to investigate in future studies to find the possible roles of phyto-components associated with *Murraya koenigii* to participate in the antibacterial potential. Also, there have been significant reports on individual nanoparticles like silver and copper oxide to suppress the growth of pathogenic bacteria [15, 33]. But there can be mere chances of developing resistance mechanisms to outgrowth the action of individual components [34, 35]. Hence developing hybrid particles are more advantageous wherein if the pathogens develop resistant to one individual particle then the counterpart can act efficiently to express the activity [36, 37].

In the present investigation, the nano-hybrid particles of silver and copper oxide displayed activity against all the test pathogens which was determined and confirmed with more than one antibacterial assays. The activity determined with well diffusion, MIC and broth dilution assay were in accordance with highest activity recorded against *P*. *aeruginosa* strain 215. Interestingly, in the present investigation the activity was found against both Gram +ve and Gram −ve pathogens with highest percentage of activity were found against Gram −ve pathogens. Based on the scientific evidence available, it can be confirmed that nanoparticles are reported to be effective antibacterial agents against wide range of test pathogens [38]. The use of nanoparticles can offer best suited alternatives as they can have multiple modes of actions [39]. Nanoparticles based on their surface charge tend to bind to the opposite charge components present in the cell wall of the test pathogens [40]. Further, the developed nano-hybrid particles and silver nanoparticles are reported to have high affinity towards thiol group of the protein which is responsible for various metabolic processes; this results in dysfunctions of the electron transport chain leading to imbalance in the physiological and metabolic processes [41].

## Conclusion

The results obtained in the present investigation revealed the role of nano-hybrid particles to express antibacterial activity against drug-resistant pathogens which were clinically isolated from myriad patients. The overall results obtained in the present investigation forms the preliminary insight on the possible role of developing nano-hybrid particles as one of the alternative resource to combat drug antimicrobial resistance.

## Data Availability

The data can be available based on reasonable request and consent from all the authors.

## Abbreviations

Ag-CuO: Silver-copper oxide
UV: Ultraviolet
FTIR: Fourier transform infrared spectroscopy
XRD: X-ray diffraction
TEM: Transmission electron microscopy
+ve: Positive
−ve: Negative
NaOH: Sodium hydroxide
*A*. *baumannii*: *Acinetobacter baumannii*
*P*. *aeruginosa*: *Pseudomonas aeruginosa*
*K*. *pneumoniae*: *Klebsiella pneumoniae*
MRSA: Methicillin-resistant *Staphylococcus aureus*
*E*. *coli*: *Escherichia coli*
CLSI: Clinical and Laboratory Standards Institute
MIC: Minimal inhibitory concentration
